# Complex Management of a Geriatric Patient With Multimorbidity: A Case Study

**DOI:** 10.7759/cureus.54604

**Published:** 2024-02-21

**Authors:** Martin D Tanhaei, Luay Demashkieh, Obaidullah Noori, Peter Eupierre

**Affiliations:** 1 Biomedical Engineering, University of California, Irvine, Irvine, USA; 2 Biomedical Engineering, Johns Hopkins University, Baltimore, USA; 3 School of Medicine, St. George's University, True Blue, GRD; 4 Internal Medicine, Loyola University Medical Center/Gottlieb Memorial Hospital, River Forest, USA

**Keywords:** interdisciplinary care, chronic kidney disease, anemia, polypharmacy, multimorbidity, geriatric medicine

## Abstract

Geriatric patients' distinct physiological responses to diseases and treatments, combined with multimorbidity and polypharmacy, make their care highly challenging. This case study examines the complex management of a 77-year-old female with multiple comorbidities, focusing on the primary issue of splenic hematoma leading to anemia. It highlights the importance of a multidisciplinary approach in geriatric care. The care she received underscores the necessity of ongoing supervision through the Bridge Model of Transitional Care, family engagement in the care process, and a customized, interdisciplinary approach to care. The conclusion of this case has implications for geriatric medicine, primary care, and specialty care, and it also influences strategies to help older adults maintain their functional status and quality of life.

## Introduction

The burgeoning elderly population globally brings to the fore the challenge of managing geriatric anemia, a condition with a found cause in approximately 80% of cases, primarily due to chronic disease and iron deficiency [1]. Anemia's prevalence in this demographic ranges from 8% to 44%, increasing notably with age and more so in men aged 85 years and older. This high prevalence and varied etiology necessitate a nuanced approach to diagnosis and management [1]. Chronic conditions such as renal insufficiency further complicate anemia's presentation and treatment, making it imperative to adopt a multifaceted management strategy. Chronic conditions like renal insufficiency significantly complicate the presentation and treatment of anemia, especially in elderly populations. Anemia associated with chronic kidney disease (CKD) is primarily normocytic and normochromic, arising from a hypoproliferative mechanism. This form of anemia is frequently linked to poor outcomes in CKD patients, including increased mortality rates [2]. The management of anemia in CKD focuses on improving renal function, when possible, and enhancing red blood cell production through the use of erythropoiesis-stimulating agents (ESAs) and iron supplementation [2]. This approach marks a significant evolution from previous treatments, which largely relied on blood transfusions and were associated with numerous complications​​ [2].

Additionally, anemia in CKD is exacerbated by the kidneys' inability to produce adequate levels of erythropoietin (EPO), a hormone essential for red blood cell production. The deficiency of EPO leads to a decrease in red blood cell count, contributing to the development of anemia [2]. This condition is particularly prevalent among individuals with kidney disease, worsening as the disease progresses and the kidneys' capacity to generate EPO diminishes further. Anemia can manifest early in the course of kidney disease and become more severe as renal failure approaches​ [2]. This case study illustrates the intricate management required for a 77-year-old female, focusing on anemia precipitated by a splenic hematoma amidst her multimorbid condition. By delving into this case, we aim to enrich the discourse on multimorbidity in geriatric care, highlighting the efficacy of an interdisciplinary approach and patient-centered decision-making in complex care scenarios.

## Case presentation

The patient, a 77-year-old female, presented to the emergency department (ED) with a chief complaint of dizziness with associated symptoms of fatigue and lightheadedness that were attributed to symptomatic anemia, against a backdrop of hypertension, gastric ulcers, hypothyroidism, hyperlipidemia, osteoporosis, and CKD. Additionally, she was treated for an asymptomatic urinary tract infection (UTI) upon admission using two doses of ceftriaxone. The first dose was given as 1 g intravenously, and the next dose was 1 g intravenous given 24 hours later.

**Figure 1:**
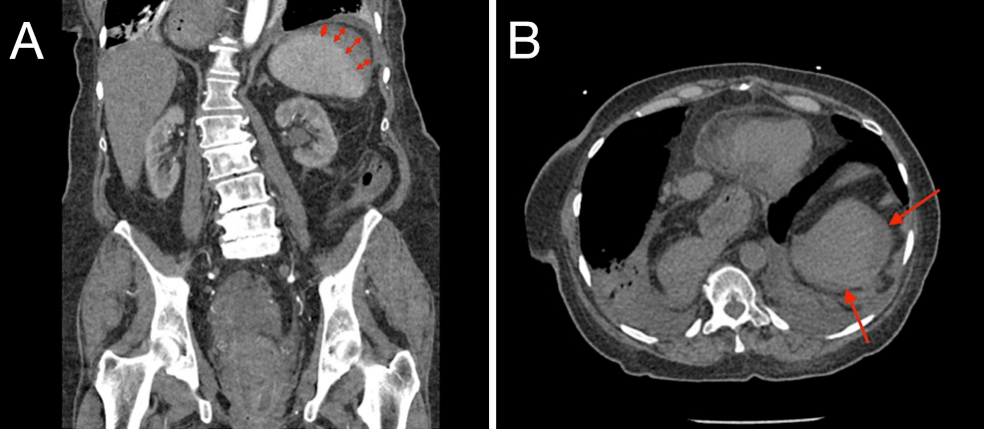
Abdominal CT scans (A, coronal and B, axial) The CT scans displayed in images A and B reveal a splenic hematoma. In Figure 1A, the double-headed arrows indicate the extent of the hematoma as seen in the coronal plane. Figure 1B uses two arrows to delineate the margins of the hematoma in the axial plane. The radiological report concluded a grade 2 splenic laceration with a peri-splenic hematoma measuring 8 cm.

Given her extensive medical history, a detailed and thorough diagnostic approach was essential. The patient's initial hospitalization was primarily aimed at identifying the cause of her acute anemia. Given her history of gastric ulcers and continued pantoprazole use, the initial diagnostic focus was on gastrointestinal (GI) causes. A colonoscopy and upper GI endoscopy revealed a hiatal hernia, diverticulosis, internal hemorrhoids, and severe vaginal prolapse, but these findings did not sufficiently explain her severe anemia, which presented with a hemoglobin level of 5.4 g/dl (Table 1). Subsequently, the CT scan of her abdomen showed a grade 2 splenic laceration with a peri-splenic hematoma measuring 8 cm (Figure 1). Adding to her diagnosis, there were small bilateral pleural effusions seen on the CT scan with no history of congestive heart failure. The patient had no recent history of trauma, thrombocytopenia, coagulation abnormalities, or abdominal pain, making the acute vs. chronic nature of the hematoma difficult to ascertain. Considering her complex medical history, a splenic hematoma was suspected as the primary cause of her anemia after common etiologies, such as GI bleeding, were ruled out. Following the diagnosis, the patient underwent four blood transfusions to manage her acute anemia effectively, stabilizing her condition and resulting in a marked improvement in her hemoglobin levels from 5.4 to 8.8 g/dl (Table 1). Due to bleeding from the complete vaginal prolapse, the care strategy was then adjusted to focus on gynecological management. The patient was then sent to have a hysteroscopy, fractional dilation, and curettage for her complete uterine prolapse. Considering her various underlying conditions, she was also suspected to be malnourished. Her BMI was normal at 24.64 kg/m^2^ and was stable throughout her hospital visits. Her albumin levels stayed within the normal range (Table 1), affirming that she was well-nourished. She is an older patient with multiple conditions, but she manages herself well, putting her in the Clinical Frailty Scale 3 range. She was found to be eventually stabilized and was stable enough to be discharged home, and she was advised to monitor for symptoms, such as worsening dizziness, and follow up with her obstetricians and gynecologists' recommendations.

**Table 1:** Laboratory studies HGB: hemoglobin; HCT: hematocrit; CR: creatinine; BUN: blood urea nitrogen; ALB: albumin; ED: emergency department visit; HS: hospital visit.

Date (visit #)/lab parameters	12/18/23 (ED #1)	12/19/23 (HS #1)	12/20/23 (HS #1)	01/01/24 (ED #2)	01/06/24 (ED #3)	Normal range
HGB (g/dL)	5.4	7.1	8.8	11.7	10.5	12 - 16
HCT (%)	18.0	22.4	26.4	37.4	33.1	36 - 47
Cr (mg/dL)	2.60	1.61	1.36	2.11	1.80	0.6 - 1.1
BUN (mg/dL)	39	18	10	18	20	6 - 20
ALB (g/dL)	4.3	3.5	3.2	4.1	3.4	3.2 - 4.8
BMI (kg/m^2^)	24.64			24.37	23.81	18.5 - 24.9

A week later, she had her second ED visit with the complaint of diarrhea associated with abdominal pain. An extensive workup was done, including a viral panel that included tests for Shiga toxin, influenza, and COVID-19, as well as blood work and urinalysis, which all revealed no acute pathology. In addition, another CT scan was done to assess the stability of the previous splenic laceration. The splenic hematoma was shown to be expanding; however, the ED physician concluded that the change was insignificant because she had no active signs of bleeding. Her hemoglobin was stable at 11.7 g/dl (Table 1). She was administered intravenous fluids and discharged home the same day. The patient continued to have signs of diarrhea for 14 days. She was presented to the ED a week later, after her second ED visit. The patient had been experiencing diarrhea every day since her last ED visit, with minimal improvements. The patient was diagnosed with dehydration, hypokalemia, and hypomagnesemia, and tested positive for *Clostridioides difficile* (CD). She was admitted, treated with IV fluids, and then discharged to complete a 10-day course of oral vancomycin. The diarrhea was resolved subsequently after the treatment, and she has improved. She subsequently developed redness and pain in her foot and went to an immediate care clinic. She was diagnosed with gouty arthritis, likely due to her acute kidney injury (AKI), secondary to hypovolemia. She was prescribed colchicine for acute symptom control and was discharged home. She was then seen by her primary care physician, where a nonsteroidal anti-inflammatory drug was considered, but due to her CKD, she was prescribed allopurinol for long-term management of her hyperuricemia.

**Figure 2:**
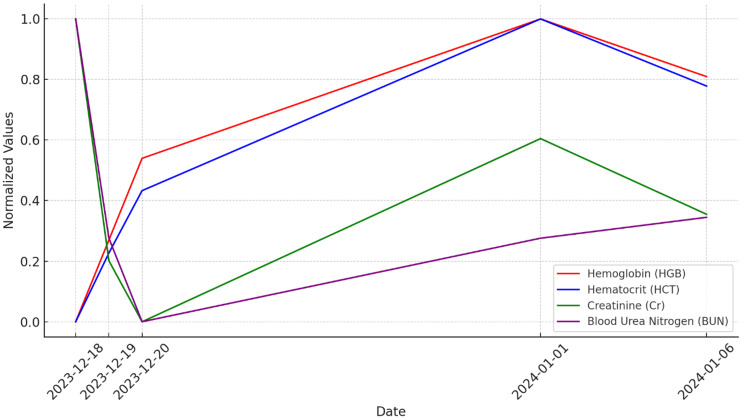
Normalized lab results over time This graph displays the normalized lab results over time using a scale from 0 to 1. Normalization was applied using the MinMaxScaler method.

These timelines of events underscore the challenge of managing acute and chronic conditions in her case during her course of treatment and the treatment strategies. Each ED visit marked a pivotal moment in the patient's care, revealing the evolving nature of her condition. For instance, her second ED visit, prompted by diarrhea and abdominal pain, extended our understanding of her health status post-initial treatment, indicating the need for ongoing vigilance and adaptability in her care plan. This visit underscored the unpredictable trajectory of geriatric patient care, where new symptoms can emerge as side effects of treatment or as independent conditions. The patient and her family's active participation in care discussions ensured that treatment decisions were closely aligned with her personal health goals and comfort, reinforcing the effectiveness of patient-centered care. A notable instance was their input during discussions about the shift to gynecological management, where their preferences and the patient's comfort were prioritized, demonstrating the value of integrating patient and family perspectives into the care process. This collaboration not only enhanced the patient's care experience but also reinforced the treatment plan's alignment with her and her family's values and expectations. This treatment strategy allowed for the normalization of her labs over time and control of her anemia (Figure 2).

Her medications included pantoprazole, ceftriaxone, furosemide, lisinopril, levothyroxine, dicyclomine, and ferrous sulfate, while the patient presents with acute anemia that is attributed to blood loss. Her treatment included a comprehensive medication regimen, necessitating careful monitoring for interactions, particularly given her renal impairment. The treatment regimen and strategy were imperative for the improvement of her renal function (Table 1). This case poignantly illustrates the complexities inherent in geriatric care, especially when managing patients with multimorbidity. The need for a dynamic, patient-centered approach is evident, as the patient's condition required constant reassessment and adaptation of the treatment plan. The case exemplified the necessity of interdisciplinary collaboration, involving specialists from gynecology, urology, gastroenterology, and beyond, to tailor her care effectively. For instance, the interdisciplinary team's decision to prioritize gynecological intervention over further invasive diagnostics was pivotal, demonstrating the value of combining insights from multiple specialties to tailor the care plan to the patient's evolving needs.

## Discussion

This case study underscores key geriatric medicine principles, illustrating the complexity of managing symptomatic anemia in older adults. By identifying a splenic hematoma as an atypical but significant cause after ruling out common etiologies such as gastrointestinal bleeding and CKD, this case enriches our understanding of anemia's diverse presentations in geriatric patients.

Anemia in older adults is a significant clinical concern, often indicating underlying pathology or exacerbating existing chronic conditions [1]. This case uniquely highlights the connection between splenic hematoma and anemia in elderly patients. Managing incidental findings in older adult patients presents a challenge, as the patient's present findings of a non-traumatic grade 2 splenic hematoma presented a treatment dilemma. The approach to treatment for geriatric patients often necessitates a careful balance between conservative management and surgical intervention, particularly given the increased vulnerability of older adult patients to complications from surgical procedures. Age-related changes in the kidneys of older adult patients predispose them to AKI from conditions such as hypovolemia, sepsis, drug toxicity, and perioperative complications [3].

In the management of our patient's multimorbidity, the challenges of polypharmacy were highlighted, particularly with the use of empirical ceftriaxone therapy for an asymptomatic UTI and proton pump inhibitors (PPIs) like pantoprazole. Despite the patient's CKD, ceftriaxone was administered without dosage adjustments due to its renal clearance characteristics [4], inadvertently leading to CD overgrowth and subsequent diarrhea by disrupting gastrointestinal biofilm [5]. The use of PPIs further elevated the risk of CD infection [6], underscoring the complexities of drug interactions and their impact on geriatric patients. The nuanced decision-making around antibiotic use for severe CD infection in patients with comorbid CKD, with considerations between fidaxomicin and vancomycin based on efficacy, patient health status, and cost-effectiveness [7,8], reflects the intricacies of managing infections in this population. Effective polypharmacy management in this context involved meticulous medication reviews, adjustments based on evolving clinical status, and careful monitoring to minimize adverse effects [9,10]. This case underscores the imperative for judicious medication management in elderly patients with multimorbidity, highlighting the critical roles of continuous care, patient and family involvement, and adaptability in optimizing outcomes.

This case highlights the effectiveness of patient-centered care and the Bridge Model of Transitional Care in managing the complexities of diagnosing and treating anemia amidst multimorbidity in the elderly. By prioritizing the patient's and family's preferences, we aligned medical decisions with her values, resulting in a tailored approach to her splenic hematoma and gynecological treatment. The holistic and interdisciplinary strategies employed, including seamless care transitions, comprehensive discharge planning, and close coordination with social workers, significantly improved the patient's quality of life and functional status [11,12]. This approach underscores the critical role of integrating patient preferences and interdisciplinary collaboration in enhancing outcomes for geriatric patients with complex care needs.

## Conclusions

This case report of a 77-year-old patient with multimorbidity, including a non-traumatic splenic hematoma leading to anemia, underscores the necessity of a flexible, patient-centered, and interdisciplinary approach in geriatric care. It demonstrates the importance of integrating patient and family preferences and fostering collaboration across specialties to improve outcomes. The case exemplifies the balance needed between conservative management and timely treatment adjustments based on changing clinical conditions.

It showcases the Bridge Model of Transitional Care's effectiveness in handling complex cases by emphasizing tailored care strategies that respect patient values and ensure interdisciplinary cooperation. The case suggests that future research should aim to enhance diagnostic accuracy and treatment effectiveness for geriatric patients with intricate needs, focusing on care plans that are both medically robust and aligned with patients' life goals and preferences.
